# Does host socio-spatial behavior lead to a fine-scale spatial genetic structure in its associated parasites?

**DOI:** 10.1051/parasite/2019062

**Published:** 2019-11-07

**Authors:** Elodie Portanier, Mathieu Garel, Sébastien Devillard, Jeanne Duhayer, Marie-Thérèse Poirel, Hélène Henri, Corinne Régis, Daniel Maillard, Elizabeth Redman, Christian Itty, Patricia Michel, Gilles Bourgoin

**Affiliations:** 1 Université de Lyon, Université Lyon 1, CNRS, Laboratoire de Biométrie et Biologie Evolutive UMR 5558 69622 Villeurbanne France; 2 Office National de la Chasse et de la Faune Sauvage, Unité Ongulés Sauvages 5 allée de Bethléem, Z.I. Mayencin 38610 Gières France; 3 Department of Comparative Biology and Experimental Medicine, University of Calgary, Faculty of Veterinary Medicine CA-T3B 2C3 Calgary Canada; 4 GIEC du Caroux-Espinouse Fagairolles 34610 Castanet-Le-Haut France; 5 Université de Lyon, VetAgro Sup, Campus Vétérinaire de Lyon 1 Avenue Bourgelat BP 83 69280 Marcy l’Etoile France

**Keywords:** *Ovis gmelini musimon* × *Ovis* sp., Host-parasite co-structure, Population genetics, Nematode, Mouflon, *Haemonchus contortus*

## Abstract

Gastro-intestinal nematodes, especially *Haemonchus contortus*, are widespread pathogenic parasites of small ruminants. Studying their spatial genetic structure is as important as studying host genetic structure to fully understand host-parasite interactions and transmission patterns. For parasites having a simple life cycle (e.g., monoxenous parasites), gene flow and spatial genetic structure are expected to strongly rely on the socio-spatial behavior of their hosts. Based on five microsatellite loci, we tested this hypothesis for *H. contortus* sampled in a wild Mediterranean mouflon population (*Ovis gmelini musimon* × *Ovis* sp.) in which species- and environment-related characteristics have been found to generate socio-spatial units. We nevertheless found that their parasites had no spatial genetic structure, suggesting that mouflon behavior was not enough to limit parasite dispersal in this study area and/or that other ecological and biological factors were involved in this process, for example other hosts, the parasite life cycle, or the study area history.

## Abstract (fr)

Les nématodes gastro-intestinaux, et plus particulièrement *Haemonchus contortus,* sont cosmopolites et pathogènes chez les petits ruminants. Étudier leur structure génétique spatiale est aussi important que d’étudier celle des hôtes pour pleinement comprendre les interactions hôtes-parasites et les processus de transmission. Pour les parasites ayant des cycles de vie simples (par exemple, les parasites monoxènes), on s’attend à ce que les flux de gènes et la structure génétique spatiale dépendent fortement du comportement socio-spatial de leurs hôtes. En utilisant cinq loci microsatellites, nous avons testé cette hypothèse pour des *H. contortus* échantillonnés dans une population sauvage de mouflons méditerranéens (*Ovis gmelini musimon* × *Ovis* sp.) dans laquelle les caractéristiques de l’espèce et de l’environnement génèrent des unités socio-spatiales. Nous avons néanmoins mis en évidence que leurs parasites ne présentent pas de structure génétique spatiale, ce qui suggère que le comportement des mouflons ne restreint pas la dispersion des parasites dans cette aire d’étude et/ou que d’autres facteurs biologiques et écologiques tels que d’autres hôtes, le cycle de vie du parasite, ou l’histoire de l’aire d’étude jouent un rôle dans ce processus.

## Introduction

Parasitism has been shown to impact numerous host characteristics (e.g., survival [45], body condition [20] and behavior [23]). Studying parasite population ecology is thus crucial to better understand and predict parasite impacts on host populations. Among the diverse ecological elements to be studied to have a complete picture of parasite population ecology, population genetics is among the most important since it makes it possible to identify ecological drivers of population structure, helping to gather information about processes often difficult to observe directly in parasite species (e.g., species, such as dispersal or demographic changes [34]). Due to their lifestyle, panmixia of a parasite population (and thus random spatial distribution of allelic frequencies) can be disrupted by the non-random transmission of parasites between hosts [34]. Spatial genetic structures of parasite populations are thus expected to rely on different hosts characteristics (e.g., spatial behavior and dispersal) [15, 41, 62].

Ruminants can host a large diversity of external and internal parasite species, especially in their digestive tract [71, 76]. Gastrointestinal parasites are major parasites of ruminants, due to their high prevalence and potential impact on their host fitness and population dynamics, in both domestic (e.g., cattle *Bos taurus* [11] and sheep *Ovis aries* [69]), and wild ruminants (e.g., Soay sheep *Ovis aries* L. [16, 40]). The most prevalent gastrointestinal parasites are often the Coccidia and nematodes (e.g., in roe deer *Capreolus capreolus* [2], Mediterranean mouflon *Ovis gmelini musimon* × *Ovis* sp [14], see also [44] for a review, African buffalo *Syncerus caffer* [29], zebra *Equus quagga*, springbok *Antidorcas marsupialis*, blue wildebeest *Connochaetes taurinus*, gemsbok *Oryx gazella* [44]). Among the diverse abomasal species of parasites identified, the nematode *Haemonchus contortus* is a widespread and pathogenic parasitic worm of small domestic and wild ruminants (e.g., mouflon [44, 52, 65, 82], chamois *Rupicapra r. rupicapra*, roe deer, Alpine ibex *Capra ibex ibex*, domestic goat *Capra hircus* and sheep [9], African buffalo [6]). Studying population ecology of this parasite, including population genetics, is of prime importance for a better understanding and management of its impact on host populations. While most studies on the genetic structures of *H. contortus* populations were performed on livestock (see [9, 72]), revealing low levels of genetic differentiation even at large (e.g., state or country) spatial scales (reviewed by [33]), scarce knowledge is available in wild populations [15]. In such populations, the behavioral ecology of the host species (e.g., philopatry, sexual segregation, dispersal or migration) and landscape structure and connectivity influence host movements (e.g., [42, 83]) and may generate marked socio-spatial structures at small intra population scales (e.g., [53, 64]). However, how these host populations’ structures influence the gene flow of their parasites is still an open question for numerous populations of wild ruminants [15].

We aimed here to help answer this question by studying the spatial genetic structure of a *H. contortus* population parasitizing an isolated wild Mediterranean mouflon population. In this population, males and females have been shown to be spatially structured (Supplementary Data 1 and [32, 57, 66]) and to have stable home ranges from year to year (see [54], Appendix S2 in [55]). Because *H. contortus* has a direct life cycle (monoxenous parasite), for which free-living infesting larvae remain close to the host feces [59], we expected the socio-spatial behavior to generate a spatial genetic signature in the population of *H. contortus*.

## Materials and methods

**Figure 1:**
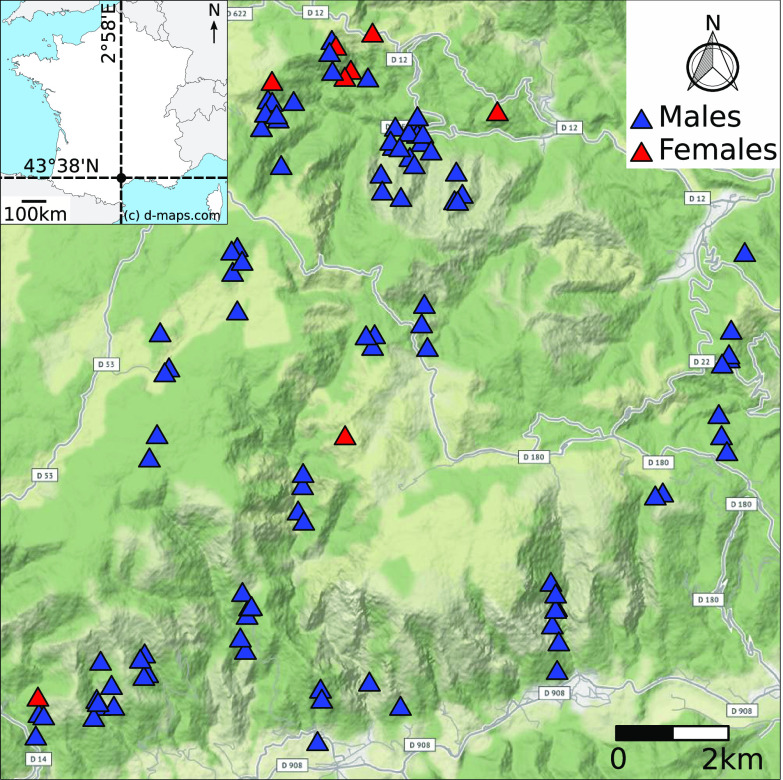
Map of the Caroux-Espinouse massif and locations of the *Haemonchus contortus* sampled (blue triangles: males, red triangles: females). Random spatial noise of a few meters using a uniform distribution was introduced to avoid obtaining duplicate coordinates for parasites sampled in a same host. The map was generated with the *ggmap* R package [49]. France country map© d-maps.com (https://d-maps.com/carte.php?num_car=2818&lang=en).

Parasites were sampled from the abomasum of 85 Mediterranean mouflon harvested between September 2011 and February 2012 in the Caroux-Espinouse massif (43°38′N, 2°58′E, 17,000 ha, 130–1124 m a.s.l, southern France, Fig. 1). Morphologically identified *H. contortus* [74] were present in 70.5% of individuals (based on an aliquot of the abomasum content: estimated median number of worms by parasitized individuals = 39, min = 3, max = 1100). For genetic analyses, a total of 115 adult *H. contortus* (107 males and 8 females, Fig. 1) were sampled from 43 mouflon (33 males and 10 females, all but one older than 4 years, i.e., adult individuals having a fixed home range [24, 25, 27]) so that a mean of 2.67 *H. contortus* were sampled by host (min = 1, max = 7).

DNA was extracted from 5 mm of the body of each individual, sampled in the head extremity, and avoiding female genital cords and hence egg DNA contamination. We used the EZ-10 Spin Column genomic DNA Minipreps Biobasic kit (ref BS628). Following supplier recommendations but adjusting volumes to the small size of samples, the lysis was performed in 100 μL of ACL buffer and 7 μL of proteinase K. Samples were incubated for 1 h at 55 °C under agitation (400 rpm). Purification and the two washing steps were performed using 150 μL of AB solution and 200 μL of buffer for each washing. Elution was done using 50 μL of EB buffer.

For each sample, seven microsatellites (*Hcms25, Hcms27, Hcms33, Hcms36, Hcms40, Hcms22co3* and *Hcms8a20*, see [63, 68], three multiplexes, see Supplementary Table 1) were amplified through polymerase chain reaction (PCR) in a final volume of 15 μL composed of QIAGEN Multiplex PCR kit Mastermix (ref. 206145), 40 nM of each primer, and 2 μL of DNA solution. PCR cycles consisted of 15 min of activation (95 °C), followed by 40 cycles of denaturation (30 s, 94 °C), annealing (1 min 30 s at primer-specific annealing temperature, see Supplementary Data 2) and extension (1 min, 72 °C). Cycles were followed by a final extension step (30 min, 60 °C). PCR products were resolved on a capillary sequencer ABI 3730XL (Thermo Fisher Scientific) by the Genoscreen laboratory (Lille, France). The electropherograms obtained were analyzed using GENEMAPPER software (Applied Biosystems/Life Technologies) and read by two independent analysts to determine allele sizes for each individual and markers. This microsatellite panel is known to produce unambiguous genotypes and to be highly polymorphic, and has demonstrated its relevance in previous *H. contortus* genetic structure studies (e.g., [68]).

Genotyping errors were tracked using MICROCHECKER v.2.2.3 software [78]. Using FSTAT v.2.9.3.2 software [35, 36], we determined genetic diversity indices (see Table 1) and tested for departures from Hardy–Weinberg (HW) equilibrium and linkage disequilibrium between pairs of loci (none detected, results not shown). Observed heterozygosity (*Ho*) was determined using R software (R core team 2016), and the hierfstat package [37].

**Table 1:** Number of alleles sampled (*N*_*a*_), allelic richness (*A*_*R*_), observed heterozygosity (*Ho*), expected heterozygosity (*He*) and *Fis* values (bold values are significantly different from zero, adjusted Bonferroni nominal levels: 0.01 [3]) for the five loci included in the population genetics analyses of the *Haemonchus contortus* sampled in the Caroux-Espinouse massif.

Locus	*N*_*a*_	*A*_*R*_ a	*Ho*	*He* b	*Fis*	*Fis p*-values
*Hcms22Co3*	5	5.00	0.31	0.52	**0.40**	0.01
*Hcms25*	15	14.93	0.78	0.84	0.08	0.04
*Hcms33*	5	5.00	0.54	0.58	0.07	0.16
*Hcms36*	8	7.96	0.73	0.67	−0.10	0.96
*Hcms40*	10	9.96	0.44	0.72	**0.39**	0.01
Mean ± *SD*	8.6 ± 4.16	8.57 ± 4.13	0.56 ± 0.20	0.67 ± 0.12	**0.17 ± 0.22**	–

a Calculated using the rarefaction method [28].

b Sensu Nei’s gene diversity [61].

The spatial population genetic structure of *H. contortus* was first investigated using a sPCA (spatial Principal Component Analysis, library *adegenet* of *R* software) [47, 48, 60]. We performed eigenvalue tests (*n* = 9999) to assess the significance of the local and global spatial structures [60]. The connection network was set using the inverse of the Euclidean pairwise distances between individuals. We then ran 10 independent runs of the MCMC simulations implemented in GENELAND v.4.0.8 software [39], using the correlated allele frequencies and the null allele models (see Results) to test for *K* varying from 1 to 10, with 1,000,000 iterations, a thinning of 100, and a burn-in of 1000. All analyses involving R packages were conducted with R 3.3.2 (R core team 2016).

## Results

Of the seven loci selected for genotyping, one (*Hcms8a20*, Supplementary Data 2) failed to amplify and was thus not included in the dataset. MICROCHECKER indicated higher than 0.05 null allele frequencies for the loci *Hcms22Co3, Hcms27* and *Hcms40* (*f* = 0.18, 0.29 and 0.20, respectively, Van Oosterhout et al.’s estimator, [78]). In addition, 28% of the sampled individuals failed to be genotyped at the Hcms27 locus which was thus excluded from the dataset. The five remaining loci considered in subsequent analyses showed a relatively high level of genetic diversity (Table 1). For all loci, the *Fis* value was 0.16 and significant (*p* = 0.01), suggesting overall deviation from Hardy–Weinberg equilibrium likely attributed to the high null allele frequencies observed for some loci [19].

The sPCA revealed no significant global (*p* = 0.97) or local (*p* = 0.32) spatial genetic structures when considering the first positive and negative axes (Supplementary Data 3). Accordingly, in the 10 independent GENELAND runs, the maximum posterior density was obtained for *K* = 1 (Supplementary Data 3), indicating an absence of spatial genetic structure in the study area.

## Discussion

In the present study, we hypothesized the socio-spatial behavior of Mediterranean mouflon (Supplementary Data 1, [32, 57, 66]) to limit *H. contortus* gene flow, resulting in a significant genetic structure in the parasite population. We, however, did not evidence any spatial patterns in the distribution of parasite genetic variability. An absence of genetic differentiation, even at large spatial scales, has already been described for nematodes in wild host populations [1, 72]. Several explanations can be proposed to explain such a result in the study area.

First, gene flow of parasite species such as *H. contortus* having an environmental phase in their life cycle might be maximized even if their hosts are only slightly mobile, because this allows for parasite exchanges without a need for the hosts to encounter one another, but just to share feeding areas. In addition, male mouflon perform reproductive excursions during the rutting period (Marchand et al., unpublished data [66]) and, even though this is not systematic, young males might disperse [26] and act as super-spreaders [51, 79] due to higher shedding rates than adults (e.g., Bourgoin et al. unpublished data, [80]). Finally, although Mediterranean mouflon are spatially structured, some overlap persists between socio-spatial unit home ranges (see Fig. 1 in [56], Supporting information G in [66]). Taken together, these host behavioral characteristics might favor step by step parasite exchanges between sub-populations of hosts, and be sufficient to ensure parasite gene flow across the entire study area.

Second, *H. contortus* is a generalist parasite of small ruminants [75], and roe deer (*Capreolus capreolus*) inhabit our study area. Although roe deer are present at much lower densities than those for Mediterranean mouflon [4], they may contribute to *H. contortus* gene flow by having different socio-spatial behaviors such as territoriality [43], attraction for forest edges [70], or marked dispersal abilities [21], linked to their parasitic infestation [22]. In addition, while currently only low numbers of domestic ruminants (~300 sheep and 300 cattle, French Ministry of Agriculture and Food 2000) are reared in the massif, mainly in the outlying areas of the mouflon range [5, 17, 18], domestic sheep and cattle were historically present at high densities in the study area before Mediterranean mouflon introduction (60 years ago). *H. contortus* might thus also have been present before the mouflon introduction, and the current spatial genetic structure of *H. contortus* could result from the historical and contemporary interactions between these three different hosts’ spatial structures and behaviors.

We encountered methodological issues with null alleles that may also raise questions about our statistical power for detecting spatial genetic structure in the parasite population. However, null alleles tend generally to induce an overestimation of genetic structure rather than the opposite, and only interfere slightly with assignments of individuals to genetic clusters [7, 10]. Since we observed no genetic structure, we were therefore confident that the presence of null alleles here led to conservative results. In addition, the sPCA uses allele frequencies as variables [47] and numerous alleles were identified in the five loci studied here (see Table 1), consequently increasing the statistical power to detect genetic structure, even though only a limited number of loci are involved. The relatively high null allele frequencies observed reflected the high genetic diversity reported in *H. contortus* worldwide and in the present study, in which the number of alleles by locus is even higher than the number observed in other populations (see e.g., [68, 81]). Null alleles have frequently been reported in other studies on *H. contortus* (e.g., [12, 13, 46, 68]) and more generally on parasitic nematodes (e.g., [38, 73]). This can be explained by the high effective population sizes (one host can carry thousands of worms) characterizing parasitic worms, favoring rapid evolution of DNA sequences and thus mutation in the flanking region of microsatellite loci [10, 63].

## Conclusions and perspectives

Contrary to our expectations, we did not detect any spatial genetic structure in *H. contortus* parasitizing Mediterranean mouflon of the Caroux-Espinouse massif. This result highlights that studying both sides of host-parasite interactions is crucial to fully understand and predict the sanitary evolution of populations, since parasite dispersal is often the result of more than one ecological factor [58]. It also reveals that results about the impacts of host spatial behavior on parasite transmission (e.g., [41]) might be difficult to generalize to diverse host-parasite systems. Specific studies on given host-parasite systems are thus needed to conclude about parasite population ecology and impacts of host ecology on their evolution. It is especially important for parasites having an indirect transmission process such as *H. contortus* or for wild hosts being in contact with domestic animals since these biological characteristics might interact with host ecology and increase the spatial scale at which parasite transmission occurs (e.g., [8]). In the current global context of habitat fragmentation [30], which has the potential to impact host population structures, genetic diversity and thus fitness [31, 67], the results of the present study also demonstrate how the overall impact of changes on populations should be assessed at the community level, since different species, such as hosts and parasites, might be differently impacted. To go further in the understanding of *H. contortus* population ecology, an interesting perspective could be to resample *H. contortus* in the different host species (mouflon, roe deer and domestic ruminants) present in the study area to determine whether the panmictic population we detected in Mediterranean mouflon also extends to other sympatric species. Such a comparison would help us to understand how parasites spread in the Caroux-Espinouse massif and give supplementary information to wildlife managers when defining management and conservation planning.
