# Supplementary material for: Does host socio-spatial behavior lead to a fine-scale spatial genetic structure in its associated parasites?
Source: Parasite. 2019 Nov 7;26:64. doi: 10.1051/parasite/2019062 (PMC6836744; doi:10.1051/parasite/2019062)
Supplement: Supplementary file 1 [file parasite-26-64-olm.pdf]

Does host socio-spatial behavior lead to fine-scale spatial genetic structure of its associated parasites?

Elodie Portanier[1], Mathieu Garel[2], Sébastien Devillard[1], Jeanne Duhayer[1,2,5], Marie-Thérèse Poirel[1,5], Hélène Henri[1], Corinne Régis[1], Daniel Maillard[2], Elizabeth Redman[3], Christian Itty[2], Patricia Michel[4] and Gilles Bourgoin[1,5]

[1]*Université de Lyon, Université Lyon 1, CNRS, Laboratoire de Biométrie et Biologie Evolutive UMR 5558, F-69622 Villeurbanne, France.*

[2]*Office National de la Chasse et de la Faune Sauvage, Unité Ongulés Sauvages, 5 allée de Bethléem, Z.I. Mayencin F-38610, Gières, France.*

[3] *Department of Comparative Biology and Experimental Medicine, University of Calgary Faculty of Veterinary Medicine, Calgary, Alberta, Canada*

[4] *GIEC du Caroux-Espinouse, Fagairolles, F-34610, Castanet Le Haut, France*

[5]*Université de Lyon, VetAgro Sup, Campus Vétérinaire de Lyon, 1 Avenue Bourgelat, BP 83 F-69280, Marcy l'Etoile, France.*

Correspondence: Elodie Portanier, Université Claude Bernard Lyon 1, CNRS, Laboratoire de Biométrie et Biologie Évolutive, 69100, Villeurbanne, France, Fax: +33 4 72 43 13 88, E-mail: elodie.portanier@gmail.com

Orcid Elodie Portanier: 0000-0002-6566-6263

Supplementary data 1: Spatial structure of Mediterranean mouflon

Spatial data were collected in a National Hunting and Wildlife Reserve (1,658 ha, 532-1,124 m above sea level) which is situated in the central part of the Caroux-Espinouse massif (see [4], Figure 1). The Mediterranean mouflon population inhabiting the reserve has been monitored since 1974 by capture-mark-recapture. Two types of spatial data were used: (i) visual observations, since marked animals were visually monitored year-round and spatially located on a map virtually divided into 125x125 m squares and (ii) GPS data, since numerous individuals have also been equipped with GPS collars (Lotek 3300S, revision 2; Lotek Engineering Inc., Carp, ON, Canada) since 2006. GPS data were collected according to two different schedules: one recording locations intensively every 20 minutes over 48-hour periods 1-3 times per month, and one recording locations continuously every 2 hours (see [6-7] for more details).

In subsequent analyses, only adult individuals having a fixed home range (i.e. two or more year-old females and four or more year-old males, see [1-3]) were considered. For individuals followed by GPS (N = 146), we randomly subsampled 20 GPS locations by animal to avoid oversampling of a very local zone over the study area (see [10]). For individuals monitored by visual resightings, only those having at least four observations were considered in subsequent analyses (N = 290). We pooled both datasets to compute the arithmetic center of all its locations for each individual. Spatial data were thus available for a total of 436 individuals (332 females and 104 males).

To determine whether Mediterranean mouflon of both sexes show socio-spatial structuring, we performed partitioning using the medoids clustering method [5] on Euclidean distances between these averaged locations. The best partitioning solution (number of clusters presents, *k*) was chosen as the one maximizing the silhouette width of value of *k* tested. As recommended by Martins et al. [8], spatial analyses were also performed with a minimum number of 10 visual resightings per individual instead of four, leading to the same results (not shown).

An optimal value of six and eight clusters for males and females respectively was observed (see Supplementary figure 1), highlighting the existence of a marked spatial structure, even at the local scale (as compared to our entire studied area) of the Wildlife Reserve.

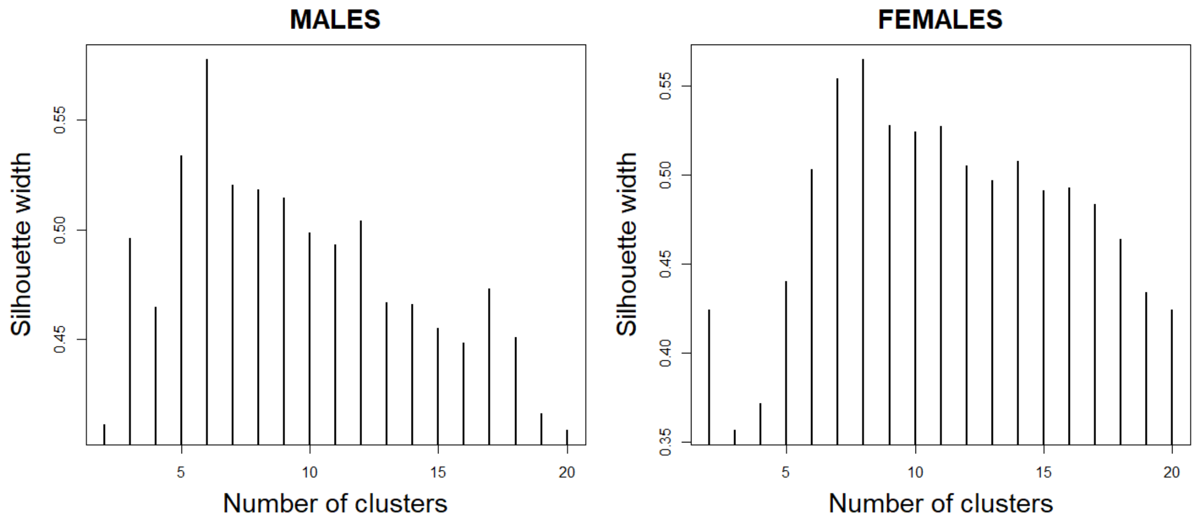

Supplementary Figure 1: Silhouette width of different clustering solutions performed on spatial data for the Mediterranean mouflon population of the Caroux-Espinouse massif.

Supplementary data 2: Microsatellite panel

Supplementary Table 1: Characteristics of the five microsatellite loci and the three multiplexes used to genotype *Haemonchus contortus* sampled in the Caroux-Espinouse massif.

Microsatellite	Primer F (5'-3')	Primer R (5'-3')	Size (pb)	Annealing (°C)	Reference	Fluorescent dyes	Multiplex	Included in main article analyses
Hcms25	ACA-GGA-GTT-ATG-AAT-TTC-CGG	GCT-TCA-GTT-TGA-ATT-GCT-TCC-C	170-312	60	[9]	FAM	A	Yes
Hcms8a20	CAA-ACT-TGA-CCC-GAC-CTC-TC	AGC-GCG-TTG-CAC-AAA-ACA-TT	170-300	60	[11]	NED	A	No
Hcms27	ACA-TAA-ATC-TAG-GTA-GGG-TAG-G	ACA-GAA-GAA-CGA-TCA-GAA-TCT-C	338-358	58	[9]	FAM	B	No
Hcms40	TCG-ATA-GTT-GTC-ACT-TCC-AA	TCG-AAT-CCT-GAG-TCT-ACC-GT	233-299	58	[11]	FAM	B	Yes
Hcms33	ATA-GCG-GTT-CGG-AGG-GGT-TTC	CCC-CGT-CAA-ATA-AAA-GGC-TAG-A	180-240	58	[9]	FAM	C	Yes
Hcms36	GCA-TAG-CGG-CAA-GGA-CGT-ATG-G	CAT-GAC-GTA-CTC-TGG-TTG-TTC-G	138-158	58	[9]	FAM	C	Yes
Hcms22co3	GAG-CTT-CAT-TGA-GAG-AAT-GGA-ATT	GGT-CCT-CAT-ATA-CGA-TCA-ACT-AA	227-258	58	[11]	NED	C	Yes

Supplementary data 3: Detailed results of spatial genetic structure assessment

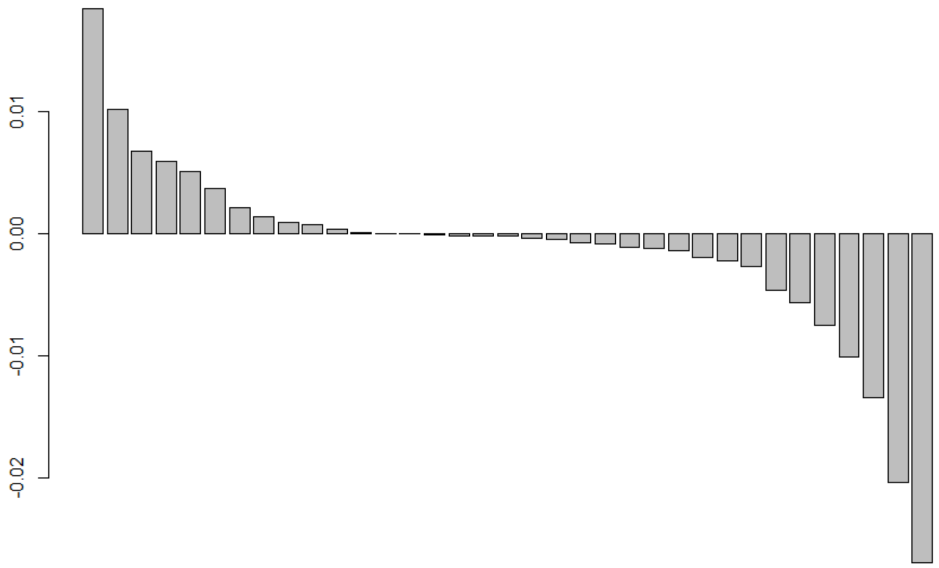

Supplementary Figure 2: sPCA eigenvalues for *H. contortus* sampled in the Mediterranean mouflon population of the Caroux-Espinouse massif.

The first positive eigenvalue was 0.018, while the next eigenvalues were lower than 0.010. The first negative eigenvalue was -0.023, while the next eigenvalues were lower than -0.020.

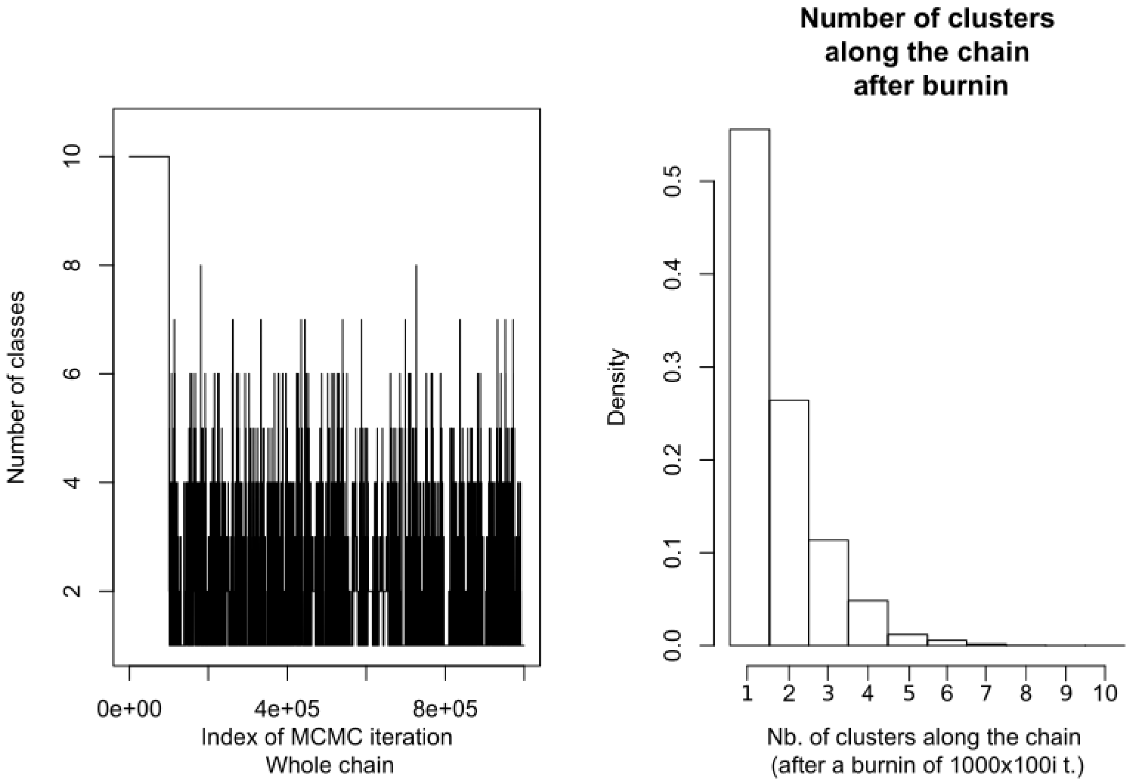

Supplementary Figure 3: Posterior density and modal number of genetic clusters inferred by GENELAND along the MCMC chain of the best run (i.e. highest posterior density) after burn in.

References

1. Dubois M, Gerard J, Maublanc ML. 1992. Seasonal movements of females Corsican mouflon (Ovis *ammon*) in a Mediterranean mountain range, southern France. Behavioural Processes, 26, 155–166.

2. Dubois M, Quenette P, Bideau E. 1993. Seasonal range use by European mouflon rams in medium altitude mountains. Acta Theriologica, 38, 185–198.

3. Dupuis J, Badia J, Maublanc ML, Bon R. 2002. Survival and spatial fidelity of moufl on (*Ovis gmelini*): A Bayesian analysis of an age-dependent capture-recapture model. Journal of Agricultural, Biological, and Environmental Statistics, 7, 277–298.

4. Garel M, Cugnasse JM, Maillard D, Gaillard JM, Hewison AJM, Dubray D. 2007. Selective harvesting and habitat loss produce long-term life history changes in a mouflon population. Ecological Applications, 17, 1607–1618.

5. Kaufman L, Rousseeuw P. 1990. Finding groups in data: An introduction to cluster analysis. John Wiley & Sons Inc. Publication, Wiley-Interscience.

6. Marchand P, Garel M, Bourgoin G, Dubray D, Maillard D, Loison A. 2014. Impacts of tourism and hunting on a large herbivore’s spatio-temporal behavior in and around a French protected area. Biological Conservation, 177, 1–11.

7. Marchand P, Garel M, Bourgoin G, Dubray D, Maillard D, Loison A. 2015. Sex-specific adjustments in habitat selection contribute to buffer mouflon against summer conditions. Behavioral Ecology, 26, 472–482.

8. Martins AG, Netto NT, Aulagnier S, Borges A, Dubois M, Vicentee L, Gerard JF, Maublanc ML. 2002. Population subdivision among mou¯on sheep (*Ovis gmelini*) ewes and ranging behaviour of rams during the rut. Journal of Zoology, 258, 27–37.

9. Otsen M, Plas ME, Lenstra JA, Roos MH, Hoelstra R. 2000. Microsatellite diversity of isolates of the parasitic nematode Haemonchus contortus. Molecular Biochemical Parasitology, 110, 69–77.

10. Portanier E, Garel M, Devillard S, Marchand P, Andru J, Maillard D, Bourgoin G. 2017. Introduction history overrides social factors in explaining genetic structure of females in Mediterranean mouflon. Ecology and Evolution, 7, 9580–9591.

11. Redman E, Packard E, Grillo V, Smith J, Jackson F, Gilleard JS. 2008. Microsatellite analysis reveals marked genetic differentiation between Haemonchus contortus laboratory isolates and provides a rapid system of genetic fingerprinting. International Journal for Parasitology, 38, 111–122.
